# Factors Affecting Total Treatment Time in Patients Treated with Orthognathic Surgery Using the Surgery-First Approach: Multivariable Analysis Using 3D CT and Scanned Dental Casts

**DOI:** 10.3390/jcm9030641

**Published:** 2020-02-28

**Authors:** Jun-Young Kim, Jin Hoo Park, Hwi-Dong Jung, Young-Soo Jung

**Affiliations:** Department of Oral & Maxillofacial Surgery, Oral Science Research Center, Yonsei University College of Dentistry, Seoul 03722, Korea; jyomfs@yuhs.ac (J.-Y.K.); wlsgnek@yuhs.ac (J.H.P.); cancer7@yuhs.ac (H.-D.J.)

**Keywords:** digital dentistry, scanned casts, orthognathic, treatment time, surgery-first approach

## Abstract

The aim of this study was to analyze factors affecting treatment times in patients treated with the surgery-first approach (SFA) for orthognathic surgery. Fifty skeletal class III patients who had undergone SFA bimaxillary orthognathic surgery were enrolled. Retrospective chart reviews and analysis of 3D CT and digitally scanned casts were conducted to assess the total treatment time. Statistical analysis was then performed with multiple study variables. Longer treatment times were required for patients with severe maxillary or mandibular teeth crowding (*p* = 0.009), a preoperative anterior open bite (*p* = 0.021), and those undergoing orthodontic extractions (*p* < 0.001). Longer treatment times were also observed when setting surgical occlusion in the postoperative anterior open bite (*p* = 0.007) and in patients with postoperative dental midline deviation (*p* < 0.001) and transverse maxillary deficiencies (*p* = 0.035). Treatment times were shorter when a class I molar key was formed in the surgical occlusion setup (*p* = 0.002) and in bilateral anterior and posterior occlusion with a minimum of four contact points (*p* < 0.001). The number of contact points, the number of extracted teeth, and postoperative midline deviation were identified as significant predictors. These results suggest that proper patient selection is important when considering SFA and that surgeons can reduce total treatment time with an appropriate surgical occlusion setup.

## 1. Introduction

The primary purpose of orthognathic surgery is to obtain stable occlusion and a positive postoperative aesthetic outcome that enhances patient quality of life. The length of treatment can drastically affect the patient’s satisfaction and overall costs [1]. However, the conventional approach of preoperative orthodontics taking place before an orthognathic surgery can exacerbate the patient’s appearance and frequently leads to a longer period of functional discomfort [2,3].

The recently advocated surgery-first approach (SFA) is reportedly beneficial as it improves facial aesthetics in a shorter treatment time compared to preoperative orthodontics. Through the regional acceleratory phenomenon, SFA can achieve efficient dental decompensation [4,5]. Preoperative orthodontics involves an alignment of teeth, incisor decompensation, and arch leveling and coordination. Surgical occlusion is more stable, as surgery is performed after arch width adjustments and/or after a correction of incisor angulation and inclination [6,7,8]. Despite these benefits, preoperative orthodontics is time-consuming, with an average duration of 24 months [9].

The SFA is challenging due to a variety of factors, such as angulation, rotation, crowding, premature contact of teeth, and arch width discrepancy. Moreover, the curve depth of Spee must be considered in advance in order to accurately predict the postoperative outcome [3]. A surgical occlusion setup is therefore a key procedure for SFA, and its setup requires considerable experience and expertise. Apart from a recent study conducted by Liao et al., quantitative research on this topic is severely lacking [10]. Moreover, many studies have only analyzed the anterior–posterior relationship as opposed to performing a three-dimensional analysis [4,11]. While most studies compare the treatment times required for the conventional approach and the SFA, none of them have examined the effects of factors for surgical occlusion setup for the reduction in the treatment time [5,12].

The aim of this study was to analyze the effects of patients’ demographic features and surgical occlusion setup on total treatment times in patients diagnosed with mandibular prognathism, who underwent surgery-first bimaxillary orthognathic surgery.

## 2. Methods

### 2.1. Study Design and Sample

This retrospective study was conducted on skeletal class III patients at the Yonsei University Dental Hospital between 2011 and 2018. The orthodontic treatment was completed after the surgery-first bimaxillary orthognathic surgery. Dental and medical charts were reviewed for preoperative demographic data collection. Patients with a history of orthodontic treatment during childhood, trauma, maxillofacial surgery, and congenital genetic deformities such as clefts were excluded. Patients who had undergone maxillary and mandibular segmental surgery were also excluded. With the exception of patients requiring surgical archwires, only those patients who had not undergone preoperative orthodontic treatment were considered. In total, 50 qualifying patients (male: 22, female: 28) were enrolled in this study. The mean age was 22.0 ± 3.4 (range: 18–34) years. To assess any differences in the total treatment time, according to the timing of the surgery, we compared the SFA group with a control group (*n* = 175). This control group was made up of patients who were treated with a conventional 3-stage approach by the same surgeon and orthodontist during the same period of time. The study was approved by the Institutional Review Board of the Yonsei University Dental Hospital (IRB No. 2-2019-0033) and the requirement for informed consent was waived due to the retrospective nature of the study.

### 2.2. Treatment Protocols

During the orthognathic surgery, LeFort I osteotomy with posterior nasal spine impaction and clockwise rotation were performed in the maxilla to correct the class III skeletal malocclusion. Intraoral vertical ramus osteotomy (IVRO) was performed for the mandibular setback. Cases were included regardless of the genioplasty requirement, depending on the chin profile. All surgeries were performed by one surgeon (YSJ). The maxilla was fixed with four L-form biodegradable fixation plates and screws (OSTEOTRANS-MX^®^, Takiron, Osaka, Japan). The mandible was not fixed during IVRO, and thus the intermaxillary fixation was performed for 10–12 days followed by active physiotherapy for approximately one month after the surgery. Postoperative orthodontic procedures were initiated after a functional recovery, with a mouth-opening of at least 40 mm and a stabilization of occlusion. The time required for surgery and active physiotherapy was included in the total treatment time. Orthodontic treatment was determined to be complete after it obtained a stable occlusion, proper alignment of teeth, dental midline alignment, and an appropriate overjet and overbite. The retention phase, initiated after completion of the orthodontic treatment, was not included in the treatment time.

### 2.3. CBCT Imaging and Preoperative Planning

All patients underwent cone-beam computed tomography (CBCT) (Alphard 3030, Asahi Roentgen Inc., Kyoto, Japan) scans approximately one month before the surgery. The data obtained from CBCT scans were converted to the Digital Imaging Communication in Medicine (DICOM) format and restructured three-dimensionally using the Simplant Pro 14.0 software program (Materialise Dental n.v., Leuven, Belgium). In addition to 3D analysis, treatment planning and surgical simulation procedures were performed.

**Figure 1:**
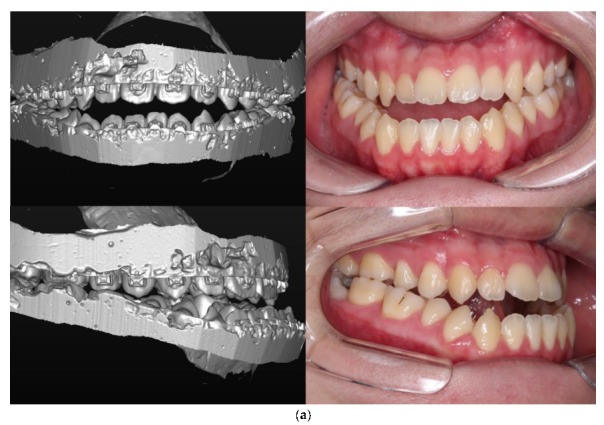

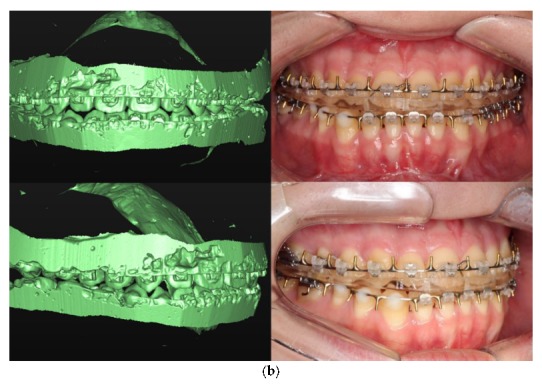
Scanned digital cast and intraoral photograph: (**a**) Preoperative occlusion, (**b**) Surgical occlusion setup and postoperative occlusion.

**Figure 2:**
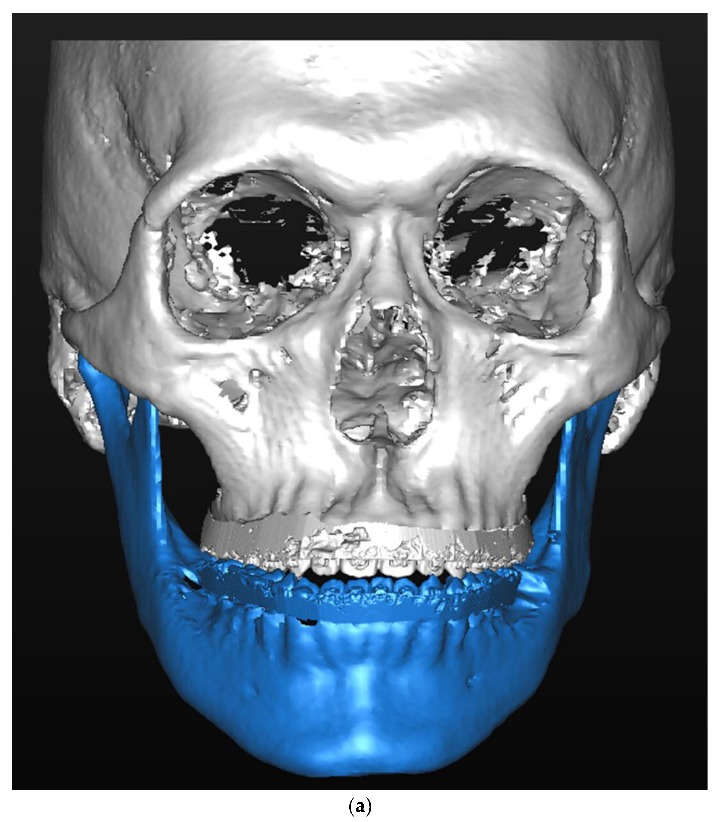

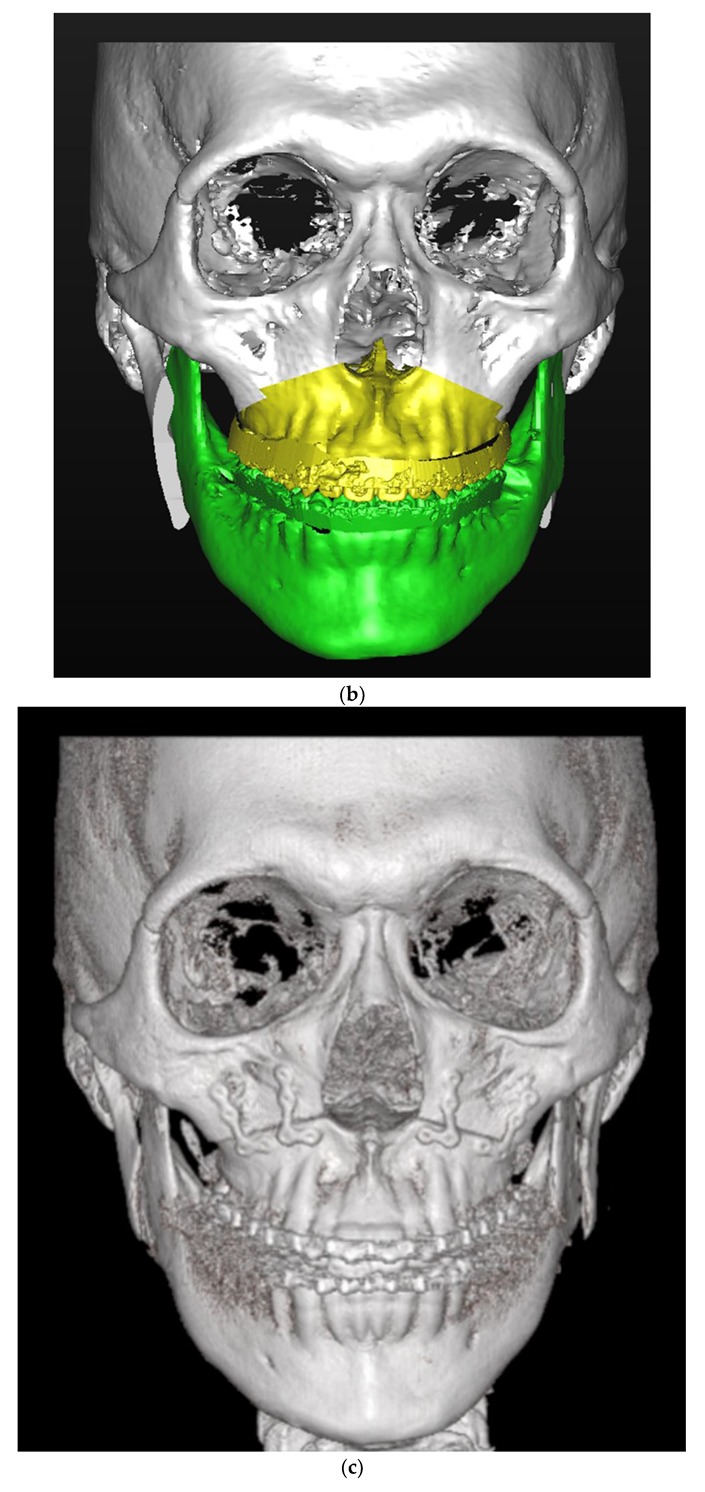

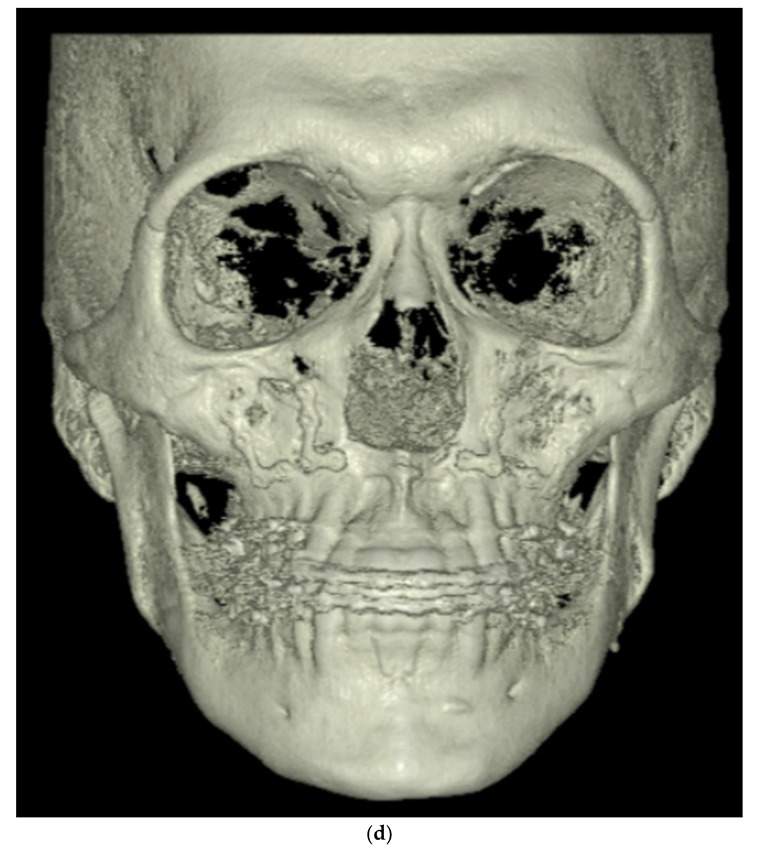
Virtual surgical simulation superimposed on a scanned dental cast: (**a**) pre-operative condition, (**b**) surgical simulation; and postoperative outcome (**c**) one month post-operatively, (**d**) one year post-operatively.

Maxillary and mandibular impressions were obtained three weeks prior to surgery in order to fabricate a dental cast and perform a dental analysis. An appropriate surgical occlusion was formed using the dental cast and a surgical occlusion analysis was performed (Figure 1). Both casts, with the preoperative and surgical occlusion setup, were scanned three-dimensionally using the Identica Blue laser surface scanner (Medit Co. Ltd., Seoul, Korea) and the STL files generated. These digital casts were superimposed on the CBCT-based 3D reconstructed skull model using the Mimics 16.0 software program (Materialise Dental n.v., Leuven, Belgium). Point superimposition was performed with a minimum of three points and the images were color-coded to ensure minimal errors during superimposition. The patterns of surgical occlusal contacts were double-checked on the dental casts and the 3D-scanned images. After superimposition, the maxilla and mandible were repositioned to a point where the skeletal discrepancy was resolved, and the surgical simulation was completed (Figure 2). Finally, surgical guides were fabricated using computer-aided design/computer-aided manufacturing (CAD/CAM).

### 2.4. Study Variables

The relationships between the treatment times, patients’ initial demographic features, and the surgical occlusion setup were evaluated.

(1) Demographic features

**Continuous variables**: Age, preoperative overbite, preoperative overjet, intercanine width, intermolar width, and total arch length discrepancy.

**Categorical variables**: Sex (female/male), facial asymmetry (yes/no), preoperative anterior open bite (yes/no), maxillary cant (mild/severe), arch length discrepancy (spacing, mild crowding, severe crowding), depth of curve of Spee (mild/severe), and orthodontic extractions of teeth (yes/no).

(2) Surgical occlusion setup

**Anteroposterior relationship**: Postoperative overjet, and postoperative molar key (class I/II/III).

**Vertical relationship**: Postoperative overbite, postoperative anterior open bite (yes/no), number of contact points, and contact types.

**Transverse relationship**: Postoperative dental midline (non-deviated, deviated) and maxillary arch expansion (not performed/performed).

In order to measure the maxillary cant, the distance between the axial plane (Frankfort’s horizontal plane) which passes through the porion and the orbitale and the mesiobuccal cusp tip of the maxillary first molar was measured bilaterally. The midsagittal plane was defined as the plane perpendicular to Frankfort’s horizontal plane, passing through the nasion and opisthion (the midpoint of the posterior margin of the foramen magnum). Deviation of menton was measured in reference to this plane, and a deviation of 4 mm or more was diagnosed as facial asymmetry.

### 2.5. Statistical Analysis

The primary investigation and outcome of this study was the treatment time. Data were preprocessed using Excel 2016 (Microsoft, Redmond, WA, USA), and the statistical analyses were performed using R (Version 3.6.1, R Project for Statistical Computing, Vienna, Austria). Statistical significance was set at *p* < 0.05. The Mann–Whitney test was used to analyze the difference between the two groups and the Kruskal–Wallis test was used to analyze the difference between the three groups. The Bonferroni post-hoc test reduced the probability of false-positive results. The effects of latent variables on the total treatment time were examined using multiple linear regression analyses. The significant variables were extracted and used in the multiple linear regression model to assess their predictive effect on treatment time. The stepwise method selected the independent variables to be included in the regression model, based on which an appropriate regression model was created.

## 3. Results

Table 1 shows the basic demographic features of the 50 SFA patients enrolled in the study. No significant difference was evident in the total treatment time according to sex, presence of facial asymmetry, and degree of maxillary cant. However, total treatment time increased by approximately five months in patients with a preoperative anterior open bite (19.0 ± 4.4 months) compared to patients without a preoperative open bite (14.1 ± 8.4 months) (*p* = 0.021). Furthermore, in terms of arch length discrepancy, treatment time was significantly increased in patients with severe crowding of 3 mm or more in the maxillary and mandibular teeth. Similarly, treatment time was increased by approximately 12.2 months in patients with orthodontic extractions (25.0 ± 7.8 months) compared to patients without extractions (12.8 ± 6.2 months) (*p* < 0.001). Treatment time increased by approximately 3.7 months in patients with a depth of curve of Spee 2 mm or more, but the difference was not statistically significant (*p* = 0.126).

Table 2 shows the effects of surgical occlusion setup on treatment time. Surgical occlusion setup can be divided into three aspects, and the results for each were as follows:

Firstly, in the anteroposterior relationship, patients with class I molar key revealed a significantly shorter treatment time compared to those with class II or class III molar key (10.8 ± 5.2 months) (*p* = 0.002). However, there were no significant differences (*p* = 0.062) between patients with class II molar key and those with class III molar key.

Secondly, in the vertical relationship, patients with a negative overbite required 18.9 ± 8.7 months for treatment completion, while patients with a positive overbite required 12.8 ± 6.8 months, which was statistically significant (*p* = 0.007). Furthermore, in terms of contact type, treatment time was significantly reduced in those patients where the bilateral anterior and posterior occlusion was formed with four or more contact points, with a mean treatment time of 8.6 ± 3.4 months (*p* < 0.001).

Thirdly, in the transverse relationship, patients with postoperative dental midline deviation required treatment times that were approximately 8.1 months longer (*p* < 0.001) and those with transverse deficiency who underwent maxillary expansion required approximately 5.2 months more treatment time (*p* = 0.035), and both these differences were statistically significant.

**Figure 3:**
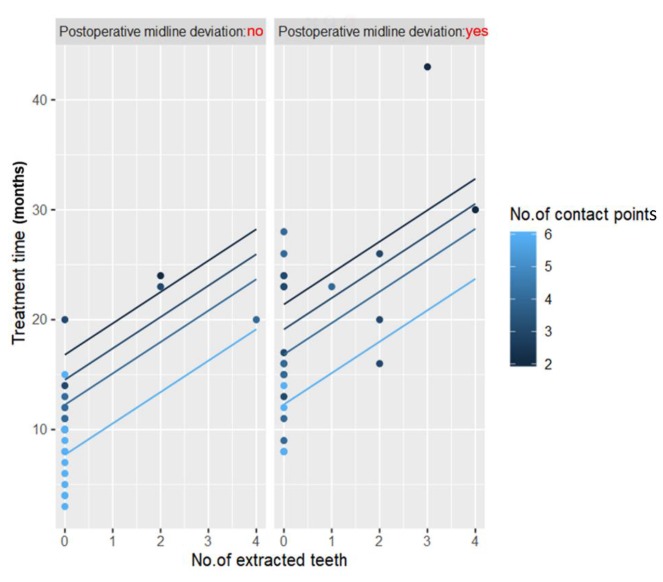
Graph showing the results of multiple regression analysis.

Using the stepwise method, three variables were selected for inclusion in the multiple regression model: the number of contact points, the number of extracted teeth, and postoperative midline deviation (Table 3 and Figure 3).

**Figure 4:**
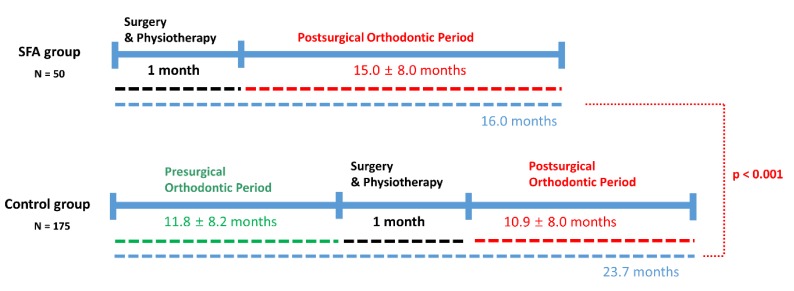
Comparison of total treatment time between the surgery-first approach (SFA) group and control group.

The total treatment time including surgery and physiotherapy was 16.0 months in the SFA group, which is a remarkable reduction compared to the control group (conventional approach) (*p* < 0.001) (Figure 4).

## 4. Discussion

Patients opting for the SFA exhibit a predilection for rapid improvements in facial aesthetics and shorter treatment duration. Since any improvement in aesthetics is already evident after surgery, SFA patients do not appreciate the long and cumbersome orthodontic treatment after the surgery. Surgeons also expect a decreased treatment time with SFA based on prior research. However, there is a lack of studies on the quantification of potential predictors of treatment time—a gap that this study aims to fill. SFA can be considered an early method of resolving skeletal discrepancy and forming malocclusion that can be treated with orthodontic treatment alone [12].

Many studies report that the total treatment time required for conventional orthognathic treatment involving presurgical orthodontics ranges from 17–36 months [9,13,14,15]. In our study, the mean treatment time in 50 patients treated by the SFA was 15.0 ± 8.0 months, which is shorter than the conventional approach.

In our previous study, we reported a total treatment time of 14 months in 37 SFA-treated patients [16]. Min et al. reported a mean total treatment time of 10.6 months, but their cases were treated by an early-surgery concept rather than SFA. Furthermore, the minimum time required by preoperative orthodontic treatment was approximately 1.1 months and that required by postoperative orthodontic treatment was approximately 8.3 months [17]. Ko et al. reported a mean treatment time of 17.8 months in patients treated by the SFA followed by orthodontic treatment in both extraction and non-extraction cases [12]. Park reported a treatment time of 20.9 months in 24 patients treated by the SFA [18]. To the best of our knowledge, this is the first study to confirm that surgical occlusion setup and differing treatment plans such as extraction or no extraction may affect treatment time in patients treated with the SFA. The results obtained in this study are significant, as no other studies have analyzed treatment times in class III patients treated with the SFA undergoing IVRO for a mandibular setback.

In the anteroposterior relation, no statistically significant association was evident between the overjet and treatment time. However, treatment time was significantly increased in patients with dental crowding and increased arch length discrepancy. As decompensating the anterior teeth after surgery is easy in most cases treated by the SFA, overjet in surgical occlusion setup is not meaningful and is determined passively, based on the degree of the anteroposterior skeletal relationship. However, as correction of overbite requires overall changes in the occlusal plane and intrusion and extrusion of several teeth, it may serve as an important factor for determining treatment time. The molar relationship is determined by the size of teeth and/or extraction or non-extraction orthodontic treatment. However, treatment time was decreased in patients in whom class I occlusion was set up compared to those with other types of occlusions setup. Nonetheless, this is expected to change depending on which premolars are extracted and clinical correlation, and therefore should assist the final decision-making process depending on the case in question [18].

In the vertical relation, our results showed that treatment time was significantly increased in patients with an anterior open bite or in those with a negative overbite in the surgical occlusion setup. Ko et al. documented that the degree of baseline overbite is an important index in determining relapse after setback surgery as well as determining the time required to achieve stable occlusion [12,19]. Yu-Fang Liao et al. argued that the posterior open bite is easier to correct than the postoperative anterior open bite and that surgical occlusion setup with an anterior open bite should be avoided. Furthermore, they stated that a posterior open bite can easily resolve malocclusion in the transverse plane [10]. We had anticipated a longer treatment time for cases with a severe depth of curve of Spee due to problems with dental alignment and arch leveling, however, no statistically significant differences were observed.

In the transverse relation, midline correction and maxillary arch expansion required increased treatment times. Transverse relation discrepancies such as maxillary arch deficiencies are occasionally observed in patients with class III malocclusion during surgical occlusion setup, but these can be countered by arch expansion using effective devices such as the skeletally anchored rapid palatal expansion. In this study, none of the cases revealed crossbites at the end of orthodontic treatment, and patients with transverse deficiencies were initially treated with maxillary expansion followed by orthognathic surgery.

Baek et al. stated that SFA should be avoided in cases involving severe crowding and/or vertical/transverse discrepancies, and that at least three stable occlusal stops and a normal positive overbite be present [3]. However, they did not perform a quantitative analysis. Several other studies on the SFA have reported relevant cases. Villegas reported the use of SFA in patients with facial asymmetry [20], and Oh et al. reported a patient with an open bite treated using the SFA [21]. Wang et al. studied the transverse dimensional changes using posteroanterior cephalograms and reported no marked differences between patients treated with the SFA and those treated with the orthodontics-first approach [22].

Finally, multiple regression analysis revealed that fewer contact points, more extracted teeth, and the presence of surgical occlusion midline deviation negatively affected the treatment times in patients treated with the SFA (Table 3, Figure 3). As the number of contact points indicates the stability of postoperative occlusion, it is an important predictor of a shortened treatment time. Several researchers suggest that at least three contact points are required for a stable occlusion [3,23], while others argue the need for a more stable posterior occlusion [5,24]. In our study, treatment time was shortened with the increasing number of contact points as opposed to the type of contact, and the treatment time decreased by approximately 10 months in patients with at least four bilateral occlusal contact points. This was an important implication that shows how increasing occlusal interdigitation could shorten treatment time. Moreover, this method can reduce severe postoperative occlusal instabilities, ultimately promoting skeletal stability [10].

We were not able to consider the impact of missed appointments on treatment time. However, patients’ social behaviors or reactions to treatment cannot be considered a shortcoming in a retrospective study. Moreover, this study had a limited sample size, and multicenter studies conducted on diverse patient groups are required.

As 3D surgical simulations are now possible, surgeons can experiment with various surgical occlusion setups and select the setup that ideally corrects the skeletal discrepancy and asymmetry. The first treatment outcome should be skeletal stability with an adequate correction of deformity; however, treatment time is an additional important factor determining the utility of treatment with the SFA.

## 5. Conclusions

Our findings suggest that initial dental status, such as dental crowding, anterior open bite, and a need for orthodontic teeth extraction, are important factors when considering the SFA. From a surgical occlusion setup perspective, class I molar key formation, proper midline and overbite setup, and at least four occlusal contacts had a positive effect on shortening the treatment time. These results may aid clinicians in diagnosis and planning for the SFA.

## Figures and Tables

**Table 1:** Effects of demographic features on treatment time.

Variables	N (%) or Mean ± SD	Treatment Time (Months)	*p*-Value
**Age (years)**	22.0 ± 3.4	15.0 ± 8.0	
**Sex**			0.147
Female	28 (56.0%)	13.1 ± 6.5	
Male	22 (44.0%)	17.3 ± 9.2	
**Facial asymmetry**			0.938
Asymmetry	28 (56.0%)	15.1 ± 8.3	
Non-asymmetry	22 (44.0%)	14.7 ± 7.7	
**Preoperative anterior open bite**			0.021 *
No	41 (82.0%)	14.1 ± 8.4	
Yes	9 (18.0%)	19.0 ± 4.4	
**Maxillary canting (mm)**			0.884
Mild (<2)	34 (68.0%)	14.7 ± 7.3	
Severe (≥2)	16 (32.0%)	15.5 ± 9.5	
**Maxilla ALD (mm)**			
Spacing (<0)	8 (16.0%)	14.1 ± 3.9	0.209 (1 vs. 2)
Mild crowded (<3, ≥0)	32 (64.0%)	12.5 ± 6.5	0.009 ** (1 vs. 3)
Severe Crowded (≥3)	10 (20.0%)	23.7 ± 9.2	0.001 ** (2 vs. 3)
**Mandible ALD (mm)**			
Spacing (<0)	5 (10.0%)	18.6 ± 13.8	0.266 (1 vs. 2)
Mild crowded (<3, ≥0)	32 (64.0%)	12.9 ± 6.9	0.387 (1 vs. 3)
Severe crowded (≥3)	13 (26.0%)	18.6 ± 6.5	0.009 ** (2 vs. 3)
**Curve of Spee (mm)**			0.126
Mild (<2)	13 (26.0%)	12.2 ± 6.4	
Severe (≥2)	37 (74.0%)	15.9 ± 8.4	
**Total ALD (mm)**	2.5 ± 2.3		<0.001 **
Non-extraction	41 (82.0%)	12.8 ± 6.2	
Extraction	9 (18.0%)	25.0 ± 7.8	
**Preoperative overbite (mm)**	−1.8 ± 2.4		
**Preoperative overjet (mm)**	0.53 ± 1.6		
**IMW difference (mm)**	5.7 ± 2.6		
**ICW difference (mm)**	8.9 ± 2.4		

* *p* < 0.05; ** *p* < 0.01. ALD: arch length discrepancy, IMW: intermolar width, ICW: intercanine width. Mann–Whitney test was performed to analyze differences between two groups, Kruskal–Wallis test was performed to analyze differences between three groups. Bonferroni post-hoc test was used to reduce the type 1 error (chances of obtaining false-positive results).

**Table 2:** Impact analysis on treatment time according to the surgical occlusion setup.

Variables	*N* (%) or Mean ± SD	Treatment Time (Months)	*p*-Value
**Anteroposterior relationship**			
**Postoperative overjet (mm)**	3.6 ± 1.6		0.378
Mild (>4)	31 (62.0%)	14.6 ± 9.0	
Severe (≤4)	19 (38.0%)	15.6 ± 6.2	
**Postoperative molar key**			
Class I	25 (50.0%)	10.8 ± 5.2	0.002 ** (1 vs. 2)
Class II	15 (30.0%)	16.6 ± 6.3	<0.001 ** (1 vs. 3)
Class III	10 (20.0%)	23.0 ± 9.5	0.062 (2 vs. 3)
**Vertical relationship**			
Postoperative overbite (mm)	0.9 ± 1.6		
**Postoperative anterior open bite**			0.007 **
No	32 (64.0%)	12.8 ± 6.8	
Yes	18 (36.0%)	18.9 ± 8.7	
**No. of contact points**	4.3 ± 1.4		
**Contact type**			
Bilateral anterior and posterior (>4 points)	18 (36.0%)	8.6 ± 3.4	<0.001 ** (1 vs. 2)
Bilateral anterior and posterior (≤4 points)	20 (40.0%)	18.3 ± 8.4	<0.001 ** (1 vs. 3)
Bilateral posterior	12 (24.0%)	19.1 ± 6.3	0.470 (2 vs. 3)
**Transverse relationship**			
**Postoperative dental midline**			<0.001 **
Non-deviated	26 (52.0%)	11.1 ± 5.6	
Deviated	24 (48.0%)	19.2 ± 8.2	
**Maxillary expansion**			0.035 *
Not performed	38 (76.0%)	13.7 ± 7.8	
Performed	12 (24.0%)	18.9 ± 7.7	

* *p* < 0.05; ** *p* < 0.01. Mann–Whitney test was performed to analyze differences between two groups, Kruskal–Wallis test was performed to analyze differences between three groups. Bonferroni post-hoc test was used to reduce the chances of obtaining false-positive results.

**Table 3:** Total treatment time prediction regression model through multiple regression analysis.

Model (Adjusted R^2^ = 0.79)	Unstandardized Coefficient		Standardized Coefficient			Collinearity Statistics	
	B	SE	Beta	*t*	*p*	Tolerance	VIF
(constant)	21.21	3.37		6.29	<0.001 **		
No. of contact points	−2.22	0.64	−0.39	−3.46	0.001 **	0.66	1.53
No. of extracted teeth	2.87	0.78	0.38	3.67	0.001 **	0.78	1.29
Postoperative midline deviation (yes/no)	4.50	1.56	0.29	2.88	0.006 **	0.82	1.21

** *p* < 0.01. The stepwise method selected the independent variables to be included in the regression model. B: regression coefficient; SE: standard error; VIF: variance inflation factor.
